# Hemorrhagic cardiac tamponade complicating myocardial infarction in a hemodialysis patient with essential thrombocythemia: A case report

**DOI:** 10.1097/MD.0000000000044898

**Published:** 2025-09-26

**Authors:** Chia-Yi Lin, Po-Jen Hsiao, Jenq-Shyong Chan, Wen-Fang Chiang

**Affiliations:** a Department of Internal Medicine, Division of Cardiology, Tri-Service General Hospital, National Defense Medical University, Taipei, Taiwan; b Department of Internal Medicine, Division of Nephrology, Taoyuan Armed Forces General Hospital, Taoyuan, Taiwan; c Department of Internal Medicine, Division of Nephrology, Tri-Service General Hospital, National Defense Medical University, Taipei, Taiwan; d Department of Life Sciences, National Central University, Taoyuan, Taiwan.

**Keywords:** cardiac tamponade, coronary artery disease, essential thrombocythemia, hemodialysis, hemopericardium

## Abstract

**Rationale:**

Hemopericardium, the accumulation of blood within the pericardial sac, can lead to fatal cardiac tamponade if untreated. Its subtle presentation often obscures classic tamponade signs, complicating timely diagnosis. Essential thrombocythemia (ET), a chronic myeloproliferative neoplasm, predisposes patients to both thrombotic and hemorrhagic complications. While aspirin is effective in reducing thrombotic risks associated with ET, it can also precipitate life-threatening bleeding, such as hemorrhagic cardiac tamponade.

**Patient concerns:**

A 71-year-old hemodialysis-dependent woman with ET experienced worsening dyspnea and chest tightness 1 month after starting dual antiplatelet therapy. This therapy was initiated immediately following a percutaneous coronary intervention for an acute non-ST elevation myocardial infarction.

**Diagnoses:**

Computed tomography revealed a massive pericardial effusion, and echocardiography confirmed cardiac tamponade. Ultrasound-guided pericardiocentesis yielded hemorrhagic fluid, establishing the diagnosis of hemorrhagic cardiac tamponade.

**Interventions:**

Pericardiocentesis resolved the effusion, but recurrent non-ST elevation myocardial infarctions and in-stent restenosis necessitated repeated balloon angioplasty.

**Outcomes:**

Discontinuation of aspirin and the addition of cilostazol to clopidogrel failed to prevent further complications. The patient later developed pulse ventricular tachycardia in the emergency department, and after her family declined cardioversion, she died.

**Lessons:**

This case exemplifies the complex challenges of managing thrombotic and hemorrhagic risks in ET patients with cardiovascular disease and renal impairment, highlighting the necessity for tailored therapeutic strategies and further research.

## 1. Introduction

Hemopericardium is the accumulation of blood within the pericardial sac. Clinically indistinguishable from pericardial effusion, it can progress to cardiac tamponade, a potentially fatal condition if left untreated. Due to its often insidious onset, patients may be asymptomatic, and classic signs of cardiac tamponade (Beck triad: hypotension, jugular venous distention, muffled heart sounds) might be absent, making early diagnosis challenging.^[1]^ Hemopericardium can arise from various etiologies, including malignancy, tuberculosis, chest trauma, myocardial infarction with free wall rupture, aortic dissection with retrograde bleeding, and complications of cardiac procedures.^[2]^ While uremic pericarditis leading to hemopericardium was once common, its incidence has significantly decreased with improved renal replacement therapies.

Antiplatelet therapy can predispose to hemopericardium, particularly in patients with recent cardiac surgery or procedures or underlying hemostatic disorders. Essential thrombocythemia (ET), a myeloproliferative neoplasm characterized by excessive platelet production, increases the risk of both thrombosis and hemorrhage.^[3]^ Despite the benefits of aspirin in ET patients with thrombotic risk factors, it can also precipitate major, potentially life-threatening bleeding. This report presents a case of hemopericardium with cardiac tamponade in a hemodialysis patient with ET who developed acute myocardial infarction and subsequently received dual antiplatelet therapy (DAPT).

## 2. Case report

A 71-year-old woman presented to the emergency department with a 1-week history of progressively worsening dyspnea and chest tightness. Her medical history included hypertension, hypothyroidism, and end-stage renal disease secondary to diabetic kidney disease, for which she had undergone regular hemodialysis for 10 years. She also had a history of coronary artery disease, managed with clopidogrel due to peptic ulcer disease. Her hypertension, type 2 diabetes mellitus, and hypothyroidism were well-controlled with telmisartan, amlodipine, bisoprolol, sitagliptin, insulin, and levothyroxine. Family history was unremarkable for thrombotic or bleeding disorders.

Three years ago, she was diagnosed with ET, confirmed by bone marrow morphology and molecular analysis revealing an *MPL* W515L mutation (JAK2 and CALR mutations were negative). Hydroxyurea therapy was initiated, stabilizing her platelet counts at approximately 500 × 10^9^/L. Due to her intermediate risk for thrombosis, presence of cardiovascular risk factor, and peptic ulcer disease, she continued receiving clopidogrel.^[4]^ One month prior to admission, acute non-ST elevation myocardial infarction (NSTEMI) was diagnosed, and she underwent percutaneous coronary intervention (PCI) with drug-eluting stents placement for stenosis of left main coronary artery and left anterior descending artery. Aspirin was added to her medication regimen.

**Figure 1.**
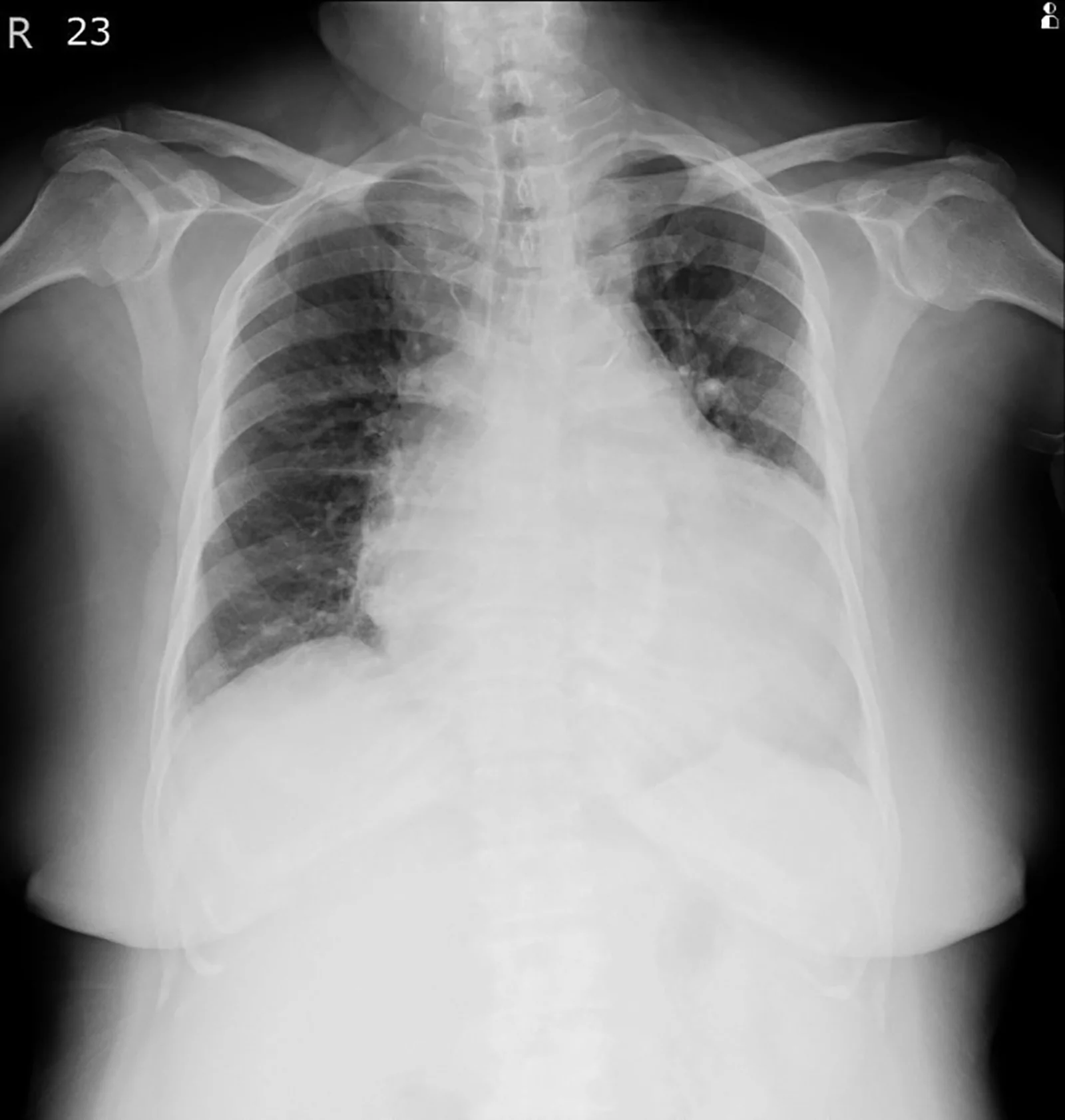
Chest radiograph demonstrating the characteristic “water-bottle” silhouette of the heart.

**Figure 2.**
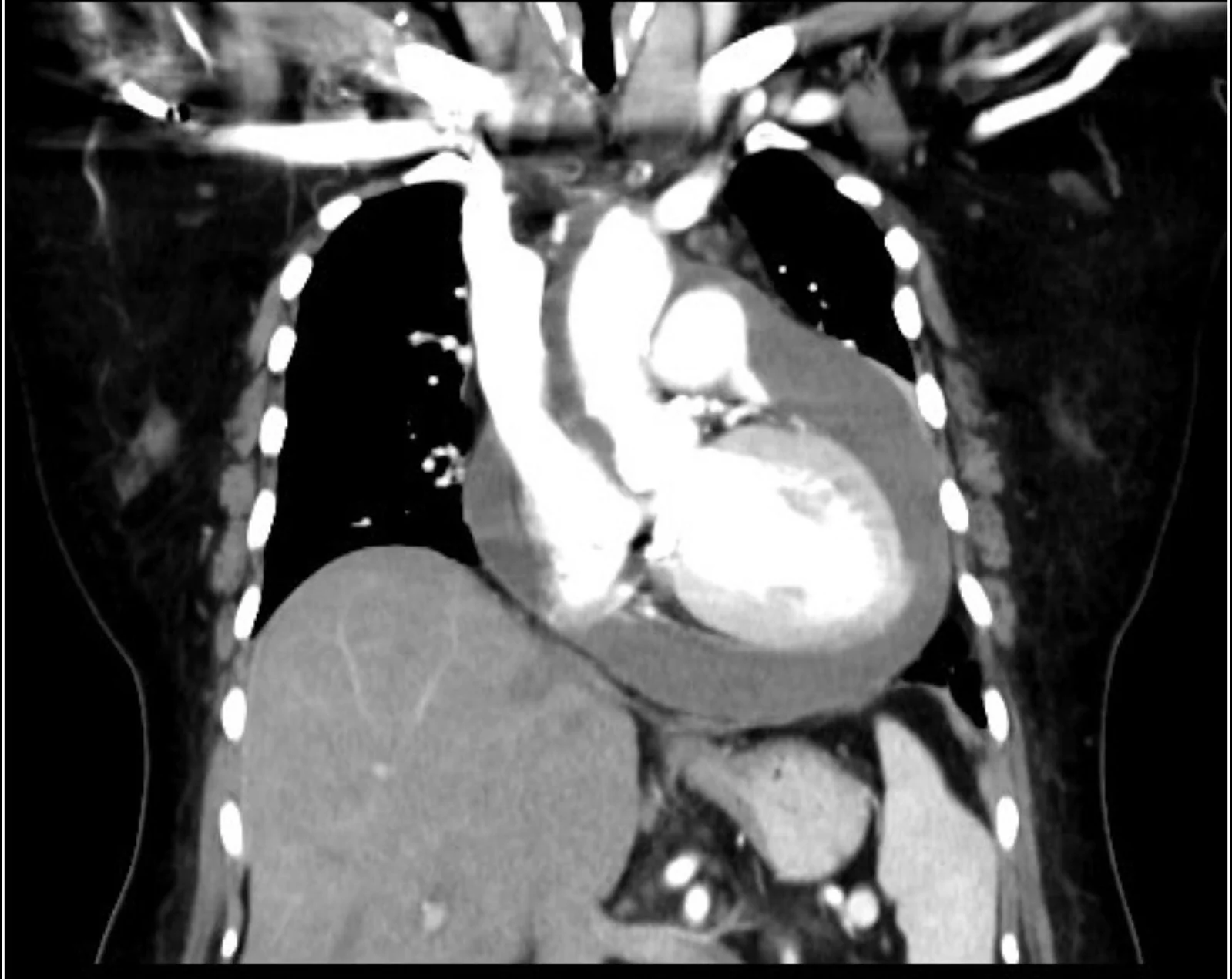
Computed tomography of the chest revealing a massive pericardial effusion.

On examination, the patient appeared fatigued but was arousable. Vital signs were as follows: temperature 35.5°C, pulse 69/min, blood pressure 118/56 mm Hg, respiratory rate 15/min, and oxygen saturation 96% while she was receiving oxygen by nasal cannula at a rate of 3 L/min. Physical examination revealed distant heart sounds and cold extremities. Laboratory studies showed a platelet count of 720 × 10^9^/L and coagulation studies were within normal limits (Table 1). Electrocardiogram demonstrated low-voltage QRS complexes without evidence of ischemia. Chest X-ray revealed a water-bottle configuration of the heart (Fig. 1). Subsequent chest computed tomography confirmed a massive pericardial effusion (Fig. 2). Echocardiography demonstrated a large pericardial effusion (>2 cm), diastolic right ventricular collapse, and a plethoric inferior vena cava with minimal respiratory variation, consistent with cardiac tamponade.

**Table 1:** Laboratory data.

Variable	Unit	Reference	Hospitalization			
			1st	2nd	3rd	4th
WBC	μL	4800–10,800	14,530	13,500	17,210	4840
Hemoglobin	g/dL	12–16	8.4	8.5	9.1	8.6
Platelet	μL	130000–400,000	720,000	649,000	819,000	408,000
PT	s	9.4–12	10.8	10.3		11.9
INR		0.8–1.2	1.02	1.04		1.2
PTT	s	25.3–32.3	27.8	25.5		37.4
Sodium	mmol/L	136–145	133.7	128.2	134.9	135.9
Potassium	mmol/L	3.5–5.1	3.1	4.5	4.2	3.4
Total calcium	mg/dL	8.6–10.2		9.5	9.7	9.2
Phosphorus	mg/dL	2.7–4.5		4.5	5.4	
Magnesium	mg/dL	1.7–2.6		2.4	2.4	1.9
BUN	mg/dL	6–20	19.3	73.8	65	33.8
Creatinine	mg/dL	0.6–0.9	2.42	7.62	8.38	3.97
AST	U/L	9–32	192	29.1	25	37.8
ALT	U/L	7–31	186	11.9	10.5	18
CRP	mg/dL	< 0.5	8.68	0.53	0.57	1.01
CK	U/L	26–308	75	148	168	121
CK-MB	U/L	<3.1			5.1	3.3
Tropoinin-I	pg/mL	<15.6	15.9	5488	6813	180.6
Total protein	mg/dL	6.5–8.1	6.0			
Albumin	mg/dL	3.1–4.8	3.2	4.2		3.9
LDH	U/L	135–225	302			
Total cholesterol	mg/dL	<200		105	101	
Triglyceride	mg/dL	<200		119	169	
LDL-C	mg/dL	<100			34.2	
HDL-C	mg/dL	>40			34.2	

ALT = alanine aminotransferase, AST = aspartate aminotransferase, BUN = blood urea nitrogen, CK = creatine kinase, HDL-C = high-density lipoprotein cholesterol, LDH = lactate dehydrogenase, LDL-C = low-density lipoprotein cholesterol.

The patient underwent ultrasound-guided pericardiocentesis, yielding 800 mL of hemorrhagic fluid. An additional 1400 mL of hemorrhagic fluid was drained over the next 3 days. Aspirin was discontinued during hospitalization. Analysis of the pericardial fluid revealed a red blood cell count of 3363 × 10^6^/L, total protein of 5.95 g/dL, lactate dehydrogenase of 1125.1 U/L, and glucose of 96.8 mg/dL. Cultures for bacteria, fungi, and acid-fast bacilli were negative. Cytological examination did not identify malignant cells. A repeat echocardiogram on hospital day 6 confirmed resolution of the pericardial effusion. Platelet function analyzer-100 closure time including collagen-EPI and collagen-ADP both showed prolonged results. The patient was discharged home on hospital day 20 with clopidogrel monotherapy.

Six months after the 1st hospitalization, the patient was diagnosed as acute NSTEMI. Laboratory data are shown in Table 1. Coronary angiography revealed left main coronary artery and left anterior descending artery in-stent stenosis, which was treated with drug-eluting balloon angioplasty. Given the concern of increasing risk of bleeding in the patient, cilostazol was added to clopidogrel as DAPT. Eight months after the 2nd hospitalization, the patient encountered another episode of acute NSTEMI. Repeat coronary angiography revealed left main coronary artery and left anterior descending artery in-stent restenosis, which was treated with drug-eluting balloon angioplasty. Twelve months after the 3rd hospitalization, she experienced sudden loss of consciousness. At emergency department, electrocardiogram showed pulse ventricular tachycardia. Considering her multiple comorbidities, her family members decided against cardioversion and the patient expired.

## 3. Discussion

ET is a myeloproliferative neoplasm marked by persistent thrombocytosis, defined as a platelet count of 450 × 10^9^/L or higher, and an increased number of mature megakaryocytes in the bone marrow. This chronic clonal stem cell disorder has an incidence ranging from 1.2 to 3.0 per 100,000 population per year and its onset in the 5th to 6th decade of life. Diagnosis typically involves a bone marrow biopsy after ruling out secondary causes of thrombocytosis. The etiology of ET is predominantly genetic, with most patients exhibiting mutations in the *JAK2*, *CALR*, or *MPL* genes. Specifically, 60% of patients have the *JAK2* V617F mutation, 20% have *CALR* mutations, and 3% have *MPL* mutations.^[5]^

ET is associated with an increased incidence of both arterial and venous thrombosis, including ischemic strokes, myocardial infarction, pulmonary embolism, peripheral arterial occlusion, hepatic or portal vein thrombosis, and deep vein thrombosis. Approximately 10% of ET patients develop myocardial infarction, often in the presence of additional cardiovascular risk factors.^[6]^ The pathogenesis of thrombosis in ET is multifactorial and involves several mechanisms that contribute to an increased risk of thrombotic events. An international collaborative study identified age > 60 years, thrombosis history, cardiovascular risk factors (tobacco use, hypertension, or diabetes mellitus), leukocytosis (≥11 × 10^9^/L), and the *JAK2*V617F mutation as risk factors for arterial thrombosis.^[7]^ In 2 recent patient cohorts from Mayo and Florence, advanced age, male gender, *JAK2* mutation, hypertension, and a history of arterial thrombosis were identified as independent risk factors for arterial thrombosis-free survival.^[8,9]^ Furthermore, elevated platelet counts, hyperactive platelets, chronic inflammation, endothelial dysfunction, and clonal hematopoiesis contribute to endothelial injury, fostering a prothrombotic environment. Dialysis patients are at heightened risk for cardiovascular disease due to a combination of traditional risk factors (e.g., age, diabetes, hypertension) and nontraditional factors (e.g., fluid overload, inflammation, oxidative stress, abnormal calcium-phosphorus metabolism).^[10]^ The thrombogenic burden associated with ET exacerbates the impact of both traditional and nontraditional cardiovascular risk factors in our patient, contributing to the progression of atherosclerotic vascular disease and consequently the development of acute NSTEMI.

In addition to thrombosis, ET can cause hemorrhagic complications, affecting 3% to 37% of patients.^[11,12]^ The pathogenesis of bleeding diathesis in ET is primarily due to defective primary hemostasis, resulting from qualitative and quantitative platelet abnormalities.^[13]^ Extreme thrombocytosis (>1000 × 10^9^/L) is often associated with a higher occurrence of bleeding due to acquired von Willebrand syndrome (AvWS), which may lead to the consumption of von Willebrand factor multimers.^[3]^ Other risk factors predisposing to bleeding include previous hemorrhage, aspirin therapy, splenomegaly and leukocyte counts over 11 × 10^9^/L.^[11,14]^ Moreover, in dialysis patients, uremic platelet dysfunction, endothelial dysfunction, anticoagulant use, and anemia collectively increase the risk of hemorrhage. Our patient had no evidence of leukocytosis or splenomegaly. While her platelet count was lower than 1000 × 10⁹/L, AvWS can also occur in this setting.^[15]^ However, a definitive diagnosis of AvWS could not be made as von Willebrand factor multimer analysis was not performed. The prolonged collagen-EPI and collagen-ADP closure times may result from the combined use of aspirin and clopidogrel, AvWS or the uremic environment in our patient.^[16]^ Taken together, the use of DAPT may predispose our patient to bleeding tendency in the setting of underlying uremia and ET, consequently leading to fetal complication of hemorrhagic cardiac tamponade.

ET patients are prone to major hemorrhagic complications, particularly in the gastrointestinal and urogenital tracts, with intracranial bleeding also being a possibility.^[14]^ Minor bleeding commonly involves mucocutaneous surfaces, such as ecchymosis, epistaxis, and gingival hemorrhage. There have been reports of bleeding complications of rare anatomic sites in ET, including spinal subdural hematoma, adrenal hemorrhage, hemarthrosis, retinal hemorrhage, intramuscular hematoma, and kidney hemorrhage.^[17]^ Pericardial hemorrhage is an extremely rare bleeding complication in ET. Only 2 cases have been reported to date (Table 2). In the 1st case, a 57-year-old man with a history of stroke and ET, who was taking aspirin and hydroxyurea, presented with a large hemorrhagic pericardial effusion. He was managed with pericardial drainage, cessation of aspirin, and a switch to clopidogrel with an increased dose of hydroxyurea. He experienced no further bleeding or thrombotic complications during the following 16 months.^[18]^ In the 2nd case, a 72-year-old woman presented with spontaneous hemopericardium leading to cardiac tamponade as the initial manifestation of ET. The hemopericardium was believed to have been induced by non-steroidal anti-inflammatory drugs therapy. Non-steroidal anti-inflammatory drugs can activate platelets, interfere with platelet aggregation inhibitors, and increase bleeding risk in ET patients. She underwent pericardial drainage, but the subsequent therapy for ET and the outcome were not reported.^[19]^

**Table 2:** Comparison of 3 ET patients with hemopericardium.

	Present patient	Kayrak et al^[18]^	Deshmukh et al^[19]^
Age (yr)	71	57	72
Sex	Female	Male	Female
Past history	CAD	Stroke	Nil
	Diabetes		
	Hypertension		
Symptoms	Chest tightness	Chest pain	Chest pressure
	Dyspnea	Dyspnea	Dyspnea
Platelet count	720 × 10^9^/L	1098 × 10^9^/L	745 × 10^9^/L
Risk factor	Aspirin	Aspirin	NSAID
Cardiac tamponade	Present	Absent	Present
Treatment	Pericardiosentesis	Pericardiosentesis	Pericardiosentesis
Outcome	Recovery	Recovery	Not reported

The 2 cases highlight a key difference in management strategy. The 1st patient’s successful outcome was achieved by discontinuing aspirin and transitioning to a single antiplatelet agent (clopidogrel), in conjunction with an increase in cytoreductive therapy (hydroxyurea). This approach focused on balancing bleeding risk by reducing antiplatelet intensity while simultaneously addressing the underlying thrombocytosis. In contrast, our patient’s therapy involved a switch from 1 dual antiplatelet regimen to another (clopidogrel + cilostazol). A key distinction in the therapeutic approach was the management of the underlying thrombocytosis. The successful outcome in the prior case suggests that effective control of the underlying ET, achieved through an increase in hydroxyurea, was a crucial factor in preventing recurrent complications. In our patient, a similar escalation of ET-specific therapy was not undertaken while antiplatelet intensity was still high. This comparison highlights how our patient’s complex combination of hemodialysis-dependent renal failure and ET may have required a more aggressive strategy to control the underlying disease, in addition to changes in antiplatelet therapy.

Clinical guidelines do not specify how to manage acute coronary syndrome (ACS) in patients with ET. The optimal antiplatelet treatment and its duration for ET patients with ACS remain unclear. Personalized antiplatelet therapy has been proposed as a strategy that optimizes the balance between safety and efficacy.^[20]^ For those with high risks of both thrombosis and bleeding events as shown in our patient, de-escalating antiplatelet therapy by aspirin discontinuation after 1 month of DAPT has been shown to reduce bleeding without any trade-off of thrombotic events.^[21]^ Another approach is guided by platelet function test or *CYP2C19* genotyping. Platelet function tests help assess the effectiveness of antiplatelet therapy by measuring platelet activity. They can identify patients with high on-treatment platelet reactivity, who may be at increased risk of thrombotic events despite taking antiplatelet drugs. *CYP2C19* enzyme converts the inactive prodrug clopidogrel into its active metabolite. Patients with certain *CYP2C19* gene variants (poor metabolizers) have reduced enzyme activity, leading to lower clopidogrel activation and increased risk of adverse cardiovascular events. In patients with low responsiveness to clopidogrel, adjunctive use of cilostazol significantly improved the clinical outcomes without increasing the risk of major bleeding.^[22]^ However, these strategies may not prevent hemorrhagic cardiac tamponade in our patient because the bleeding complication occurred only 1 month following DAPT. Additionally, the addition of cilostazol to clopidogrel monotherapy following the 1st episode of NSTEMI also cannot provide long-term effect to prevent stent restenosis in our patient. This outcome underscores the unique challenges presented by our patient’s comorbidities, which were not addressed in the general studies on cilostazol. Specifically, our patient’s hemodialysis introduced a profound bleeding risk, and her underlying ET added a layer of complexity with its own unique platelet abnormalities. This case highlights the necessity of developing new strategies for antiplatelet therapy in ET patients undergoing dialysis treatment. For example, ticagrelor monotherapy has been shown to reduce the risk of bleeding without significantly increasing thrombotic events compared to ticagrelor plus aspirin.^[23]^

In ET patients, methods of revascularization previously reported include primary PCI, percutaneous transluminal coronary recanalization, and coronary artery bypass grafting.^[24]^ However, there is an increased incidence of complications such as stent thrombosis and restenosis.^[25]^ ACS patients with ET presenting with substantial thrombi in the culprit coronary lesions, aspiration thrombectomy or intracoronary thrombolysis without stent placement may be the preferred revascularization strategies. However, if there is an underlying atherosclerotic plaque in the culprit lesions, additional interventions such as balloon angioplasty or stent placement should be considered.^[24]^ Although the required duration of DAPT for bare-metal stents was shorter, which was preferable in case of hemorrhagic complications, drug-eluting stent was implanted in our patient because of the concern of multiple thrombotic risk factors. Recent guidelines on DAPT in coronary disease have shifted, emphasizing that the duration of DAPT should be dynamic and based on clinical symptoms rather than the type of stent implanted.^[20]^ In our patient’s management, the concept of dynamic DAPT was applied in response to evolving clinical events. She was initially started on standard DAPT (aspirin and clopidogrel) following PCI for NSTEMI. However, the subsequent development of worsening dyspnea and chest tightness (ultimately diagnosed as hemorrhagic cardiac tamponade) prompted a critical reassessment of her antiplatelet regimen. Aspirin was discontinued, and cilostazol was introduced alongside clopidogrel as a dynamic modification intended to reduce bleeding risk. Although this adjustment failed to prevent the patient’s final fatal complication, it reflected a real-world effort to balance the competing risks of thrombosis (from the drug-eluting stent and underlying ET) and hemorrhage (from ET, antiplatelet therapy, and uremia).

## 4. Conclusion

This case highlights the critical challenges in managing patients with ET complicated by ACS and hemorrhagic cardiac tamponade, especially in the context of hemodialysis. Thus, not only the thrombosis but also the bleeding tendency while prescribing aspirin to the patient with ET under maintaining hemodialysis, should always be considered. The unique presentation of hemorrhagic cardiac tamponade in this patient underscores the need for individualized antiplatelet therapy strategies, balancing the risks of thrombosis and bleeding. This case calls for further research into tailored therapeutic approaches for ET patients with complex comorbidities, especially those undergoing dialysis, to mitigate risks and improve outcomes.

## Author contributions

**Conceptualization:** Chia-Yi Lin, Po-Jen Hsiao, Wen-Fang Chiang.

**Resources:** Po-Jen Hsiao.

**Software:** Chia-Yi Lin.

**Supervision:** Jenq-Shyong Chan, Wen-Fang Chiang.

**Validation:** Jenq-Shyong Chan.

**Writing – original draft:** Chia-Yi Lin, Po-Jen Hsiao.

**Writing – review & editing:** Jenq-Shyong Chan, Wen-Fang Chiang.
